# Editorial: The interaction of physical activity, genetic and environmental factors on cardiovascular health

**DOI:** 10.3389/fpubh.2025.1602611

**Published:** 2025-04-29

**Authors:** Ningxin Zhang, Kuai Yu

**Affiliations:** Department of Occupational and Environmental Health, Key Laboratory of Environment Health, Ministry of Education and State Key Laboratory of Environmental Health (Incubating), School of Public Health, Tongji Medical College, Huazhong University of Science and Technology, Wuhan, China

**Keywords:** cardiovascular health, physical activity, genetic predisposition, environmental determinants, health disparities, preventive strategies

Cardiovascular disease (CVD) is the leading cause of global mortality and loss of health life expectancy (1). In China, the burden is particularly heavy, with a total of 330 million CVD patients and nearly half of all deaths attributed to CVD (2). While genetic and environmental factors significantly contribute to CVD risk (3), modifiable behaviors such as physical activity (PA) offer critical opportunities for prevention (4). Furthermore, the interplay between lifestyle behaviors and cardiovascular health remains a critical area of public health research, particularly as populations age and occupational demands evolve (5). This editorial synthesizes findings from recent studies to elucidate the interplay between genetic susceptibility, environmental exposures, and behavioral factors in shaping cardiovascular outcomes, with implications for public health policy and personalized interventions.

The Research Topic comprised four original research articles (Liu et al., Li and Zeng, Wang et al., Zhang et al.), primarily focusing on the effects of social activity, lifestyle factors, sedentary time on CVD-associated indicators, and reporting the state of PA between population of Chinese Han and Tibet ethnicity. These articles provide valuable insights into preventive measures for CVD.

Liu et al. compared the PA and physical fitness (PF) levels of Han and Tibetan children and adolescents in China. Tibetan youth exhibited higher engagement in moderate-to-vigorous physical activity (MVPA), muscle-strengthening exercise (MSE), and organized sports activities (OSP) than Han youth, potentially linked to cultural practices and high-altitude adaptations. These findings underscore the need for ethnically tailored health policies into school programs, while urban Han populations may benefit from expanded community sports infrastructure.

Li and Zeng explored the relationship between sedentary behavior and elevated blood pressure after utilizing China's statutory retirement policy as an exogenous variable, based on the five rounds of data from the China Health and Nutrition Survey (CHNS) 2004–2015. Increased leisure sedentary time post-retirement raised diastolic blood pressure (DBP) by 0.66 mmHg per hour, with higher education and familial healthcare access mitigating this effect. These results highlight the unintended consequences of retirement-related lifestyle shifts and advocate for policies promoting active aging, such as community exercise initiatives and public health campaigns targeting sedentary habits.

Wang et al. analyzed data from the Chinese Longitudinal Healthy Longevity Survey (CLHLS) and found that increased social activity participation among older adults (≥65 years) reduced 10-year all-cause mortality risk by 21–30%, though no significant association with heart disease incidence was observed. Social engagement likely confers protection through psychosocial and physiological pathways, emphasizing the need for programs fostering social connectivity, particularly among isolated older adult populations.

Zhang et al. identified liver fibrosis as a mediator between occupational physical activity (OPA) and elevated blood pressure in construction workers. High OPA intensity correlated with increased liver fibrosis indices including FIB-4 and APRI, exacerbated by smoking and alcohol use. Conversely, higher education and healthcare access attenuated risks. This underscores that while excessive strain may harm metabolic health, workplace interventions (e.g., smoking cessation programs) and socioeconomic support can mitigate adverse outcomes.

This studies collectively reveal that behavioral factors influence cardiovascular health through different pathways. Socioeconomic determinants, such as education and healthcare assess, emerge as critical moderators, highlighting the role of structural inequities. For instance, health literacy empowers individuals to adopt healthier lifestyles, while urban infrastructure can encourage PA across diverse populations. While these studies provide valuable insights, limitations include reliance on self-reported data and restricted generalizability due to population-specific focuses. Future research should incorporate objective measures such as accelerometers, population-diverse cohorts, and employ longitudinal designs to validate these findings.

The integration of behavioral, genetic, and environmental perspective is essential to address the growing burden of CVD. By prioritizing equitable policies and feasible interventions, societies can mitigate disparities and improve cardiovascular health across the lifespan.
